# Age-specific epidemiology of human leptospirosis in New Caledonia, 2006-2016

**DOI:** 10.1371/journal.pone.0242886

**Published:** 2020-11-30

**Authors:** Elise Klement-Frutos, Arnaud Tarantola, Ann-Claire Gourinat, Ludovic Floury, Cyrille Goarant

**Affiliations:** 1 Service des Médecine Interne et Maladies Infectieuses, Centre Hospitalier Territorial de Nouvelle-Calédonie, Noumea, New Caledonia; 2 Institut Pasteur de Nouvelle-Calédonie, Noumea, New Caledonia; 3 Service de Microbiologie, Centre Hospitalier Territorial de Nouvelle-Calédonie, Noumea, New Caledonia; 4 Direction des Affaires Sanitaires et Sociales, Gouvernement de la Nouvelle-Calédonie, Noumea, New Caledonia; Universita degli Studi di Parma, ITALY

## Abstract

With over one million cases worldwide annually and a high fatality in symptomatic forms, human leptospirosis is a growing public health concern for the most vulnerable populations, especially in the context of global warming and unplanned urbanization. Although the Asia-Pacific region is particularly affected, accurate epidemiological data are often lacking. We conducted an eleven-year retrospective laboratory-based epidemiological survey of human leptospirosis in New Caledonia. From 2006 to 2016, 904 cases were laboratory-confirmed, including 29 fatalities, corresponding to an average annual incidence of 30.6/100,000 and a case fatality rate of 3.2%. Over the period, there was a major shift from indirect serological diagnosis by MAT to direct diagnosis by real-time PCR, a more specific and sensitive test when performed early in the course of the disease. The systematic implementation of genotyping informed on the variety of the infective strains involved, with a predominance of serogroups Icterohaemorrhagiae and Pyrogenes. The epidemiological pattern showed a marked seasonality with an annual peak in March-April. Interestingly, the seasonal peak in children of school age was significantly earlier and corresponded to school holidays, suggesting that attending school from February on could protect children from environment-borne leptospirosis.

## Introduction

Leptospirosis is among the most common zoonoses worldwide and an increasing public health concern [1, 2]. Humans are accidentally infected by pathogenic *Leptospira* principally from contaminated soils or water, or direct contact with rodents or other reservoir mammals in rural areas or flood-prone urban slums [3]. In the tropics, leptospirosis is frequently a disease of poverty [4, 5].

The World Health Organization’s (WHO’s) Leptospirosis Burden Epidemiology Reference Group estimated that 1.03 million cases occur worldwide annually with 58 900 deaths, the incidence in the tropics being approximately 10 times higher than in temperate regions [6]. Despite this major burden, leptospirosis remains a gravely neglected disease [7]. Areas with the highest morbidity and mortality are located in the Asia-Pacific Region, the Pacific Island Countries and Territories (PICTs) facing the highest burden with as many as 150 cases/100,000 population per year in Oceania [6, 8]. Effective surveillance systems with appropriate laboratory support are urgently needed, especially in the disease-endemic, developing countries of this region [9–12]. Where implemented, the epidemiological surveillance of leptospirosis is classically based on serological data, using the Microscopic Agglutination Test (MAT). This test is poorly sensitive in the first days following disease onset, highlighting the benefits of a combination of techniques including modern molecular diagnostic tests [13].

Following an incubation period of 2 to 26 days (average 10 days), symptomatic leptospirosis generally presents as a fever of abrupt onset, rigors, myalgia and headache, and an estimated 10–20% risk of severe disease including approximately 10% fatalities [6]. Despite the efficacy of largely available antibiotics when provided early enough [14], the mortality of severe presentations and global public health costs of leptospirosis remain high. Supportive care with renal replacement therapy, artificial ventilation and blood products needed to support patients during critical complications are lacking in most affected countries [15].

New Caledonia is a French archipelago currently governed under the Nouméa Accord, located in the southwest Pacific Ocean, about 1,210 km east of Australia. Its climate is subtropical with a hot rainy season from December to March. Its population was 268,767 inhabitants in the 2014 census, 74.4% of whom lived in the South Province where the capital Noumea is located [16]. The 39% of the population who declared being part of the Melanesian Kanak community live mostly outside Nouméa, frequently in rural tribal settings. Leptospirosis is thought to have been imported with boat rats during the early days of colonization in 1853, the first reports of human leptospirosis in New Caledonia dating back to 1954. Leptospirosis became a notifiable disease in 1991 and a surveillance program of cases is routinely implemented since then. Laboratory-based diagnosis for surveillance was conducted exclusively at the Institut Pasteur of New Caledonia before being transferred to the Territorial Hospital laboratory in late 2016. We aimed to review the epidemiology of leptospirosis cases in New Caledonia in order to better inform local and regional Public Health priorities and interventions. We notably tried to evaluate possible age-specific patterns, which could inform targeted prevention strategies.

## Methods

The country surveillance system for leptospirosis relies on a notification form. This form includes demographics (age, sex, address), activities likely associated with exposure, occupation, and clinical presentation. However and although notification is mandatory, only a subset of patients were correctly informed in this system, with a large number of cases not being notified, but only declared by the laboratory after biological diagnosis. We therefore reviewed the laboratory information systems for leptospirosis cases in New Caledonia from 2006 to 2016, providing access to age, sex and laboratory results only. Data for the period January 2006 –October 2016 originated from the Institut Pasteur of New Caledonia (IPNC) laboratory database and for November-December 2016 from the Territorial Hospital Laboratory of New Caledonia (CHT). Samples originated from all medical structures: Territorial and Provincial hospitals; Peripheral health centers; Private clinics; General practitioners and private laboratories.

All leptospirosis cases and associated anonymized demographic data were included in the study. Both early molecular diagnosis using a real-time PCR from serum or urine and late serological diagnosis using MAT with a panel of *Leptospira* strains of epidemiological relevance were routinely used throughout the study period [17, 18].

Diagnosed leptospirosis cases were classified as confirmed (a positive qPCR or seroconversion in paired samples) or probable (having both a clinical presentation compatible with leptospirosis and a single MAT titer ≥ 800). The qPCR used was targeting *lfb1* [19] from 2006 to 2009 or *lipL32* from 2010 on [20]. The putative infecting serogroup was defined as the pathogenic serogroup yielding the highest titer in MAT serology. In addition, genotyping using a diagnostic PCR product sequence provided a putative serogroup for qPCR-confirmed cases [13].

Data were compiled and graphed using Excel (Microsoft Corporation, Redmond, WA, USA).

We studied classical 10-year age classes for epidemiological description, using the 2014 census data (https://www.isee.nc). Patients were also classified as “school age” if aged 3 to 16 year old, the legal ages for mandatory and free school education in New Caledonia or “non-schooling” if older. Age-specific incidence rates for these age classes could not be calculated because census data are 5-year age classes ([0–4]; [5–9], etc…). Differences were therefore studied between age groups [0–14] and 15 and older (S2 File). Mean and median values were computed using Excel. Fisher and χ^2^ tests were calculated using GraphPad Prism software (GraphPad Software, La Jolla California USA), Poisson statistics were computed using Stata (StataCorp. 2013. Stata Statistical Software: Release 13. College Station, TX: StataCorp LP).

Leptospirosis is a notifiable disease since 1991 in New Caledonia. Patients are therefore informed that their anonymized data may be used for surveillance and reporting purposes. No further ethical approval was therefore required for this registry-based anonymous retrospective study. Fatality and the age of fatal cases were provided by the New Caledonia Health Authority.

## Results

### General patterns

**Fig 1:**
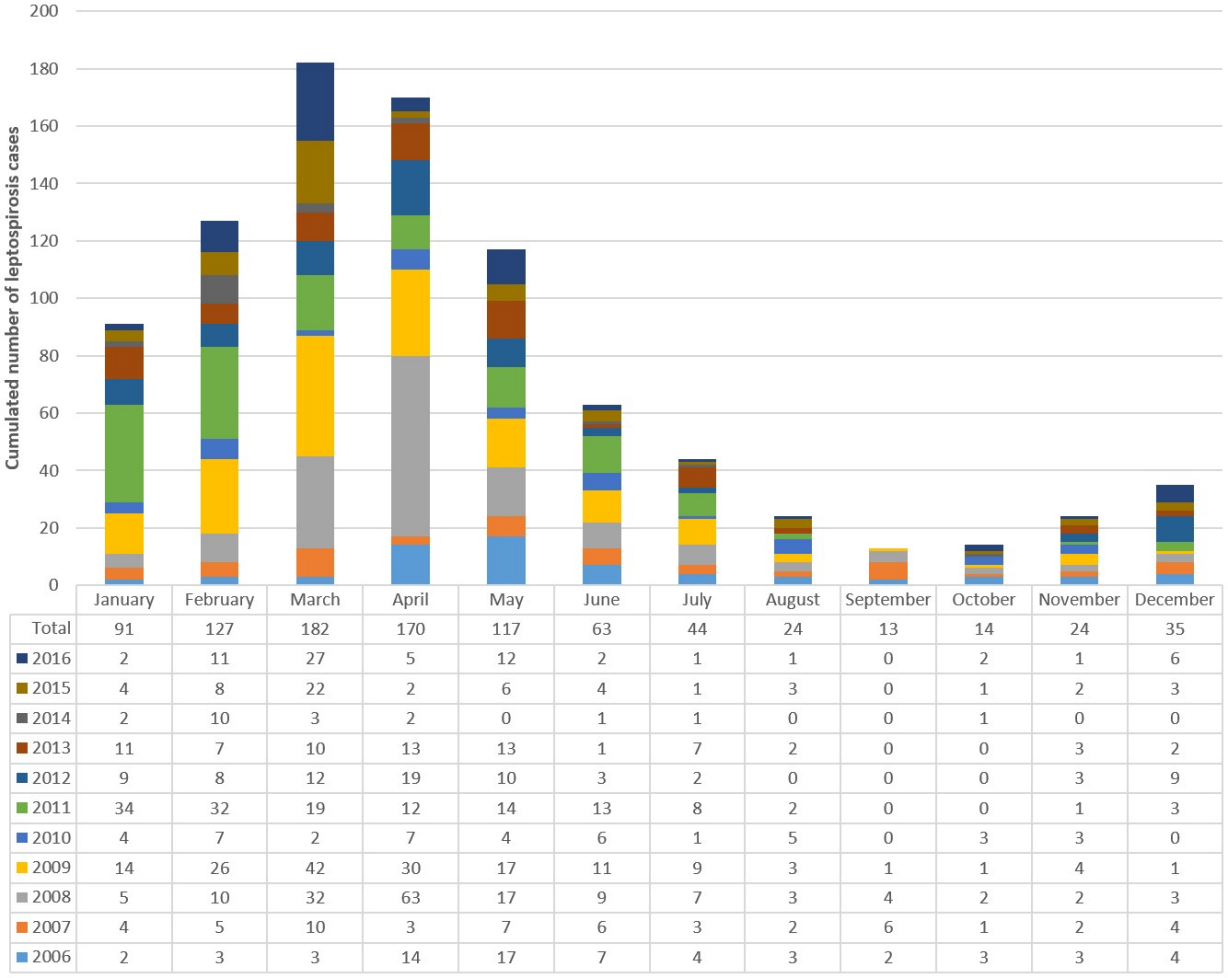
Cumulated monthly cases of laboratory-diagnosed human leptospirosis in New Caledonia during the period 2006–2016.

Between January 1^st^, 2006 and December 31^st^, 2016, 904 human leptospirosis cases were laboratory-diagnosed in New Caledonia. Of these, 700 (77.4%) were classified as “confirmed” and 204 (22.6%) as “probable”. The incidence of leptospirosis was characterized by a strong seasonal pattern, with a marked peak in incidence in March-April (Fig 1). All raw data are available in S1 File.

The mean annualized incidence rate of laboratory-diagnosed leptospirosis during the period was 30.6/100,000 population per year, ranging from 7.44 in 2014 to 59.16 in 2009. There was no overall increase but strong inter-annual variations in the incidence during the study period, with especially significant outbreaks in 2008, 2009 and 2011.

**Fig 2:**
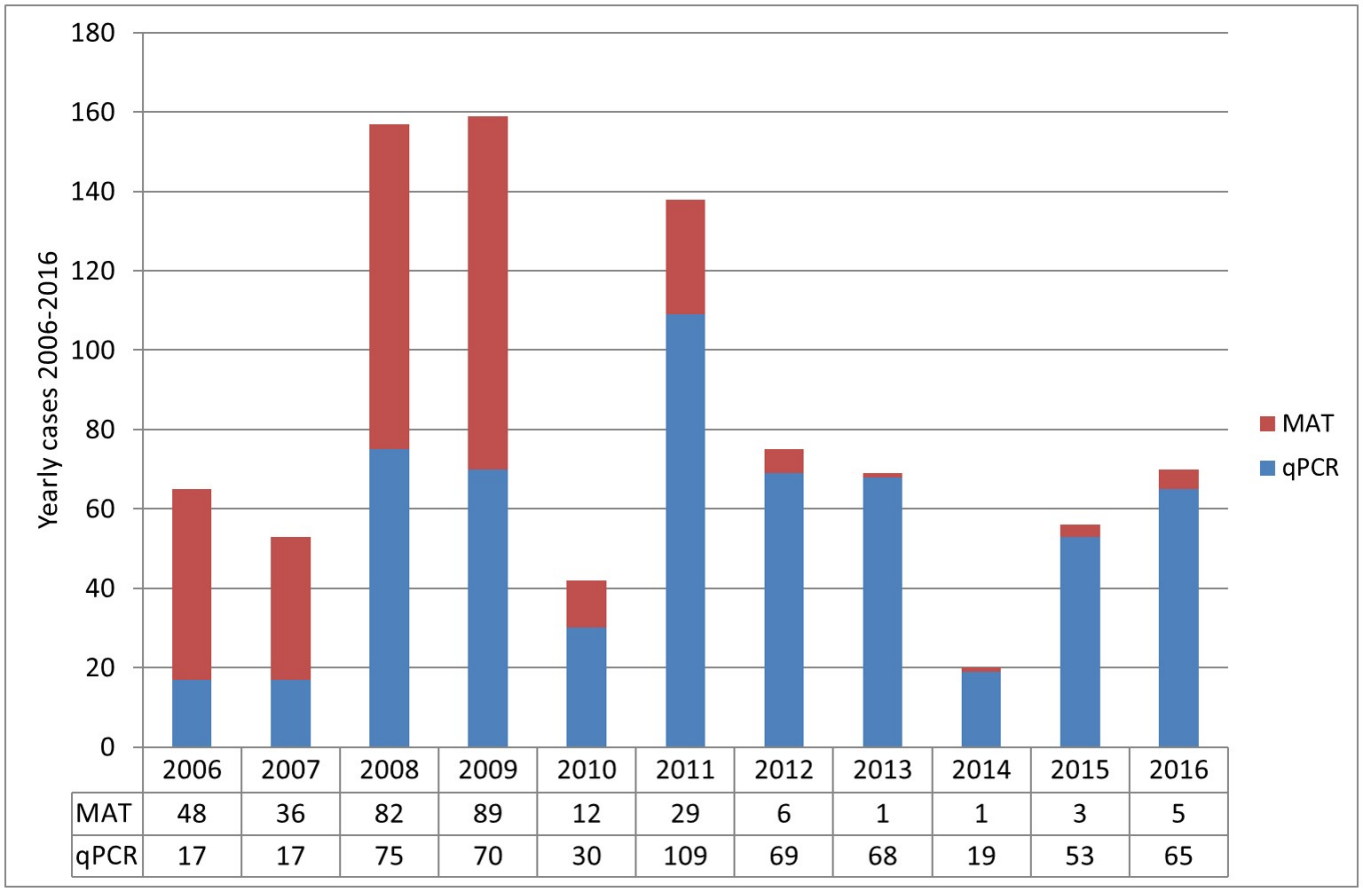
Contribution of laboratory diagnostic methods in the detection of human leptospirosis in New Caledonia from 2006 to 2016.

From the laboratory viewpoint, there was a shift in biological diagnosis between 2006 and 2016: MAT serology was the confirmation method most effective during the first years and was progressively overtaken by real-time Polymerase Chain Reaction (qPCR) from 2010 onward (Fig 2). The 2006–2009 period was associated with <50% diagnoses obtained from qPCR results, while that method ensured >70% of diagnoses from 2010 onward and >90% since 2012. This shift in diagnostic method was associated with a statistically significant increase in the percentage of confirmed diagnoses, because qPCR provides definitive confirmation. The percentage of confirmed cases rose from 267/434 (61.5%, from 2006 to 2009, when MAT was dominant) to 433/470 (92.1%, from 2010 to 2016, more than 50% of cases confirmed by qPCR) (Fisher test; p <0.001). We examined the association between the contribution of real-time PCR and deaths using lethality data from the New Caledonia Health Authority: Lethality was 12/434 (2.8%) during 2006–2009 and 17/470 (3.6%) during 2010–2016, respectively, not significantly different between the two periods (Fisher test; p = 0.57).

The sex ratio was unbalanced with 604 males: 300 females (M/F = 2.01). The median age of cases was 33.6 years (range 1.1–84.8 years). Twenty-nine patients died from leptospirosis, a mean fatality rate of 3.2% (95%CI 0.02–0.05). The mean age of fatal cases was 48.6 y.o. (median 51.7, range 17–78).

The infecting serogroup was putatively identified for 743 (82.2%) cases, by MAT serology (385 cases) and/or genotyping (449 cases). In 91 cases where both results were available, there was an agreement between serology and genotyping in all but five cases. Discordant results were all observed in early serum samples with low MAT titers. In these five cases, the genotype was used to infer an infective serogroup. In total, the serogroups involved were Icterohaemorrhagiae (426 cases, 57.3% of informed cases), Pyrogenes (148 cases, 19.9%), Australis (86 cases, 11.6%), Ballum (47 cases, 6.3%) and Pomona (12 cases, 1.6%). In addition, Panama (13 MAT results), Canicola (n = 8), Autumnalis (n = 1) and Tarassovi (n = 1) were suggested by MAT results but never confirmed by genotyping and were also never isolated in culture in New Caledonia [13, 21]. Of note, an infection by *Leptospira weilii* was known to have been acquired overseas [17]. We observed a change in this pattern over the eleven-year period with an increase in Icterohaemorrhagiae and a decrease in Canicola serogroups.

### Age-specific patterns

**Fig 3:**
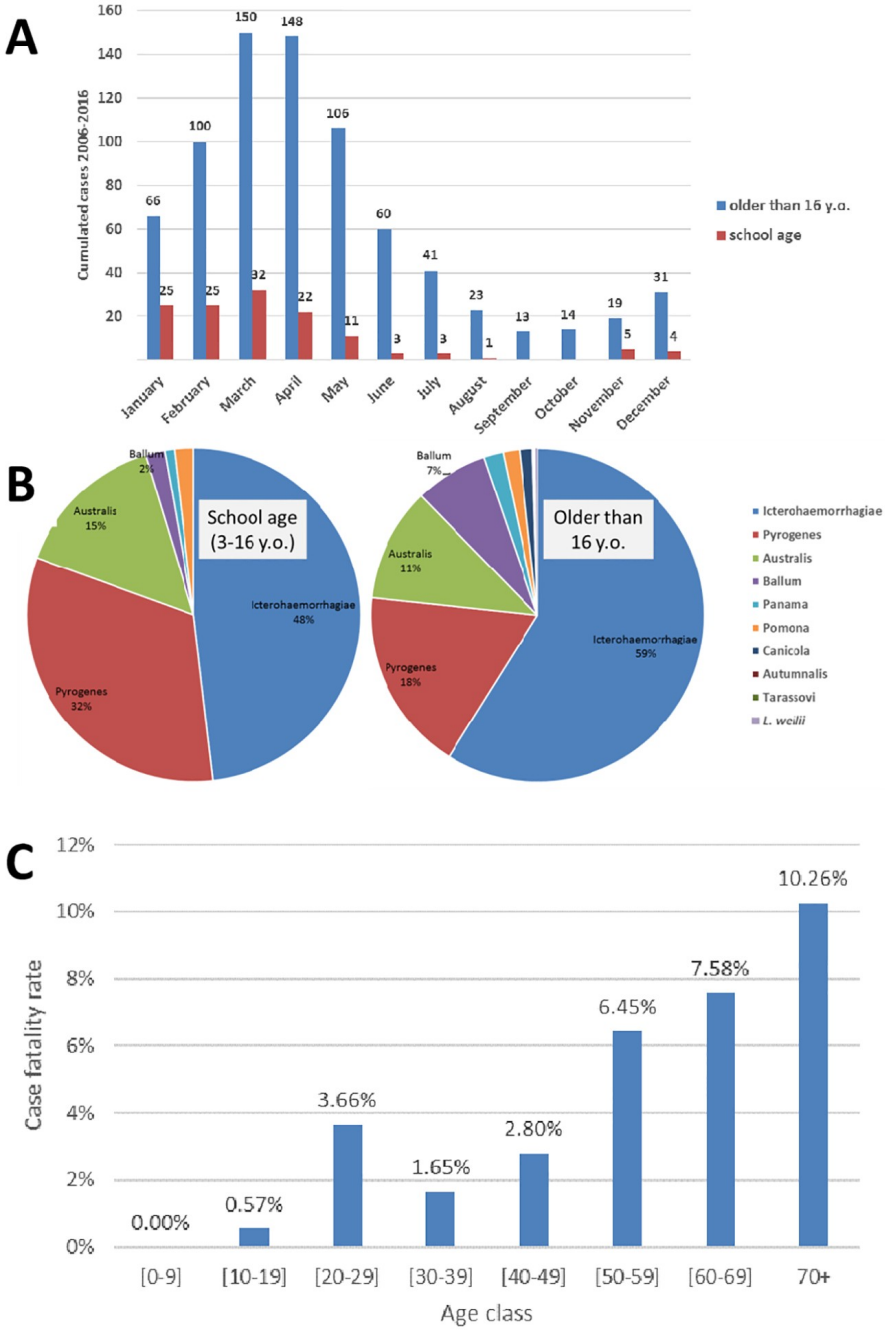
Age-specific epidemiological patterns of laboratory-diagnosed human leptospirosis in New Caledonia from 2006 to 2016. Panel A shows difference in seasonality between school age (3–16 y.o.) and older patients. Panel B shows the relative contribution of the different serogroups in documented infections (n = 743) (One Leptospira weilii infection was acquired overseas). Panel C shows the case fatality rate in the different age classes.

We investigated the age-pattern of leptospirosis during this period. The most affected population subgroup was the males aged 10–39 years with a mean annual incidence above 50/100,000 population (Table 1). There were 131 cases in the “school age” population, with a mean age of 11.7 (median 12.5). The seasonal peak of incidence in this age-group was earlier in the calendar year (Fig 3): 50 of 131 “school age [3–16]” cases (39.1%) occurred during January or February. The same two-month period accounted for only 21.5% (166 of 771) of leptospirosis cases in “non-school” age patients. To statistically evaluate this difference in seasonal incidence rates, we used census-based age classes and compared the incidence rates in patients younger than 15 year old with that in older patients. As shown in S2 File patients younger than 15 had a lower incidence rate of leptospirosis from March to December (risk ratio RR = 0.31, 95% CI [0.24; 0.40], p<0.0001) compared to older ages, but a similar incidence rate during January and February (p = 0.1197). The M:F sex ratio in school-age patient was 2.6, not significantly higher than in older ages (1.92 M/F in older people, Fisher; p = 0.13). School-age patients were also more likely to be infected with the serogroup Pyrogenes than older patients (31.8% vs 17.8%, OR = 2.126, 95% CI [1.3054; 3.4167], Fisher p = 0.0016). Of the fatal cases, more than 50% (15/29) occurred in patients aged above 50. The relative risk of fatality for patients older than 50 was 3.8203 (95% CI [1.8762; 7.7791], p = 0.0002). Age-specific patterns are illustrated in Fig 3.

**Table 1:** Number and estimated annual incidence of laboratory-diagnosed leptospirosis cases by age group and sex in New Caledonia from 2006 until 2016.

Age group (years)	N diagnosed cases			Population (2014)			Estimated annual incidence of reported cases in New Caledonia p. 100,000 pop.		
	Male	Female	Total	Male	Female	Total	Male	Female	Total
[0–9]	28	13	41	21 441	20 231	41 672	11.9	5.8	8.9
[10–19]	129	47	176	22 574	21 742	44 316	52.0	19.7	36.1
[20–29]	117	47	164	20 509	19 790	40 299	51.9	21.6	37.0
[30–39]	120	62	182	20 098	20 295	40 393	54.3	27.8	41.0
[40–49]	91	52	143	19 969	19 784	39 753	41.4	23.9	32.7
[50–59]	52	41	93	14 566	14 239	28 805	32.5	26.2	29.4
[60–69]	39	27	66	9 752	9 217	18 969	36.4	26.6	31.6
≥ 70	28	11	39	6 633	7 927	14 560	38.4	12.6	24.4
total	604	300	904	135 542	13 3225	268 767	40.5	20.5	30.6

## Discussion

Our study of all laboratory-diagnosed cases found varying annual incidences of confirmed Leptospirosis in New Caledonia over an 11-year period, with increasing confirmation due to the introduction of qPCR and an epidemiological pattern suggesting a protective effect of schooling in children. Exposure could not be studied from our data sources, but has already been reported in former articles [18], including in periods overlapping our study [22, 23].

Surveillance-based studies of leptospirosis have been conducted in French Polynesia, Hawaii, Fiji and New Caledonia, some of the few PICTs with specialized laboratories available to provide accurate data on this zoonosis [12, 24–29]. Our study in New Caledonia shows that leptospirosis has been circulating intensely over the 2006–2016 period, with a mean annualized incidence of 30.6/100,000 per year. As a comparison, 57 cases of human leptospirosis were reported in the population of 145,308 in New Caledonia between June 1983 and May 1985 (annualized incidence 19.6/100 000), and 192 during the 1989–93 period in a population of 164,173 (annualized incidence 23.4/100 000). With 239 cases and an estimated population of 225,296 the annualized incidence was 21/100,000 in the 2001–2005 period [18, 30, 31].

Comparing our results with those of the other French tropical island territories using comparable surveillance systems, the incidence of confirmed leptospirosis in New Caledonia was higher than in Guadeloupe (where it declined from 25 to 6.9 annual cases per 100,000 population in 2016), Martinique (14.2 to 6.1), Mayotte (13.1), La Réunion (11.3), or French Guyana (5.9), but lower than French Polynesia (46) or Futuna (844, the highest incidence reported worldwide) [26, 32–34]. The incidence reported in New Caledonia is almost 100 times higher than in Mainland France (or more generally in Europe) for the study period [34].

The study also found a comparatively low case fatality rate of 3.9% from 2006 until 2016, while it was 5.4% in the 2001–2005 period and 9.9% in 1989–1993 [17, 18, 31, 35]. This slow but steady decrease in lethality (χ^2^ for trend at 0.0002 and χ^2^ for linearity <0.001) suggests improved global care in New Caledonia, where lethality is now similar to that in Guadeloupe, Martinique or La Réunion [33, 34]. The case-fatality rate however was under strong influence of the age (Fig 3C), notably with no fatal cases in the school-age patients. This finding is in agreement with former reports of pediatric leptospirosis, which show that severe forms are less frequent in children [23, 36, 37]. However, because of improvements in diagnostics sensitivity arising from the use of molecular detection compared to culture [38], this apparent decrease may also reflect a higher detection rate of leptospirosis in the New Caledonia population and this trend must be interpreted with caution.

Former studies including case investigations reported important factors for human leptospirosis: Contact with rats or domestic animals; Walking barefoot in water or mud; Swimming in rivers; Gardening or farming and working in piggeries. Former studies have notably shown that the Pyrogenes strain involved in human leptospirosis in New Caledonia is most frequently acquired during freshwater activities [39]. Among 135 confirmed cases in 2008, contact with animals (OR>2), swimming, fishing and hunting (OR>3) were associated with the occurrence of confirmed cases [22, 40]. Recent findings suggest that although major animal reservoirs of leptospirosis may vary across PICTs, livestock (especially cattle and pigs), dogs and rodents may all play important roles in disease transmission to humans in the Pacific [22, 32, 41].

Our demographic findings show that men are more frequently affected, with a sex ratio of 2.01 M/F, with men in the age groups 10–39 being the most impacted. This result is in agreement with former results from New Caledonia and elsewhere [6, 12, 22, 36]. The El Niño Southern Oscillation (ENSO) has irregular cyclic recurrences. La Niña phases are associated with warmer or rainier weather in the Western Pacific, including New Caledonia, and more leptospirosis cases, likely the major contribution of the inter-annual variability in incidence observed during the period [40]. In 2008, 2009 and 2011, heavy rainfalls and floods were associated with La Niña phases of the ENSO and the occurrence of leptospirosis outbreaks [22, 40]. Our data also confirm a strong seasonality with an annual peak during March and April in New Caledonia.

The serogroups involved in human cases in our study were predominantly Icterohaemorrhagiae, Pyrogenes and Australis, with additional cases caused by Ballum and Pomona serogroups. In earlier series, cases were quasi-exclusively caused by serogroup Icterohaemorrhagiae from 1973 to 1980 [42]. Among 239 confirmed cases during the 2001–2005 period, serogroups involved were also mainly Icterohaemorrhagiae (69%), Australis (8%) and Pyrogenes (6%) [18]. We believe the fluctuations are probably not explained by the epidemiologic trends (geographical and animal origin of species) but rather by changes in the diagnostic technique. To the best of our knowledge and based on isolates [13], Panama and Canicola do not circulate in New Caledonia but are identified by poorly-specific MAT tests because these are very reactive, leading to MAT cross-reactions [21, 41]. The major contribution of serogroup Icterohaemorrhagiae is of significance, since it was shown to be associated with an increased risk of severe forms in New Caledonia [14].

Importantly, our study shows two age-specific epidemiological patterns of leptospirosis. The seasonality shift in school-age patients likely coincided with exposure during the Austral Summer school holidays (December 20 –February 15), taking the incubation period into account. Returning to school likely had a protective effect, reducing exposure during the seasonal peak of leptospirosis, in March-April, as suggested by the significantly lower odds ratio of 0.31 in children below 15 compared to older population. Our results support the hypothesis that children were exposed through freshwater activities during holidays, as also evidenced formally during source-tracking studies in the field [39]. Firstly, school-age patients (3–16 y.o.) were more frequently infected with Pyrogenes, a strain known to be most frequently associated with freshwater activities [39], a frequent leisure activity in children in the Pacific. Secondly, school-age children were infected earlier in the calendar year, suggesting that returning to school classes in February strongly decreased the infection risk in this specific population subgroup. Interestingly, a very similar pattern was observed in Japan, where young boys were infected through recreational freshwater activities during summer holidays [36]. As speculated by the authors of the study in Japan, the demographic features and exposure history reported in both studies are very similar and probably widely shared in the subtropical populations [36]. In this study conducted in the most tropical part of Japan, a high proportion of mild clinical forms was described, also in agreement with our observation of only non-fatal cases in New Caledonia.

Former studies have suggested that vaccination with a multivalent vaccine including serogroup Pyrogenes could provide a long-lasting immunity of up to 7 years [43]. Whether repeated exposure of New Caledonian children to Pyrogenes through freshwater activities provides some degree of protection in young adults would deserve to be studied.

The relative contribution of diagnostic techniques changed dramatically over the eleven-year period of our study and involved a contribution use of qPCR, especially since 2010. Real-time PCR is highly specific and can be used to diagnose leptospirosis in the first few days of the disease in a definitive way, while serological confirmation requires repeat testing which is often challenging due to the difficulty to access a convalescent serum sample from probable cases and patient loss to follow-up. During the 2008 outbreak, early diagnosis using qPCR allowed the diagnosis of 54% of the New Caledonian cases [22], a proportion that rose to more than 95% in the last years of our study period. The increase in qPCR-based diagnoses from 2006 to 2016 could optimistically suggest patients’ earlier referral or clinicians’ testing with qPCR in the blood or in the urine that could contribute to reduce the risk of complications through the timely prescription of antibiotic therapy [14, 44]. We observed a twofold decrease in lethality over the past three decades, likely resulting from earlier diagnosis, but also improvement of patients’ transfer and medical care. We found no statistical association, however, between lethality and greater qPCR use during the study period. Hopefully, accurate rapid diagnostic tests will be developed and validated and become widely available in the future [45]. Such rapid bedside tests are needed for leptospirosis, especially in tropical areas in which arboviruses, viral hepatitis, hemorrhagic fevers and malaria are common differential diagnoses.

Our study may suffer from biases and limitations. First, evolutions in diagnostic methods during the study period may have led to a bias in the number of cases diagnosed. MAT could have overestimated the number of cases in an endemic territory where repeated infections may cause antibodies to rise above detectable levels in a prolonged manner, and MAT positivity in absence of ongoing infection [46]. However, we used a ≥800 titer to define a probable case, a very high threshold that is most likely to reflect recent infection. Changes in diagnostic methods are therefore unlikely to have a significant impact on our data. Second, only patients developing severe disease are likely to be tested, leading to surveillance bias and underestimation of the true incidence. This is especially the case in rural areas, where patients may not even seek medical advice and clinical practitioners often do not resort to laboratory diagnosis in ambulatory patients and directly administer probabilistic antibiotic treatment. Although leptospirosis can be fatal, infection is most often pauci-symptomatic or benign [47, 48]. As in most other diseases, our data therefore mainly pertain to the evolution of comparatively severe cases which came to the attention of the healthcare system. It is these severe cases, however, which remain a significant public health concern in New Caledonia. Furthermore, although the efficacy of diagnostic techniques changed, the medical network and reporting system did not and the trends described largely remained unaffected. Third, we observed no significant reduction in lethality during 2006–2016 despite the increased use of qPCR. This may be due to small numbers of clinically diverse patients, leading to the difficulty to detect even strong associations. Another explanation could be that patients in the 2010–2016 period suffered from more comorbidities than patients during the 2006–2009 period, these factors having been shown to be associated with poorer outcome in untreated leptospirosis. The median age of patients, however, was comparable for the two periods, at 34.3 and 32.9 years, respectively. Finally, the shift from MAT to qPCR may have accelerated diagnostic confirmation but time to first antibiotic treatment may have remained the same due to longtime awareness of leptospirosis among clinicians, who prescribe probabilistic antibiotic treatment before obtaining laboratory confirmation. As opposed to early antibiotic treatment, there is little evidence that early diagnosis improves patient outcome; It does, however, help clinicians orient clinical management, especially in a subtropical area with several differential diagnoses. It also provides more reliable data for surveillance and timely detection of foci or outbreaks.

## Conclusion

We report an updated epidemiological description of laboratory-diagnosed leptospirosis cases in New Caledonia over a period of 11 years and analyzed laboratory diagnostic changes. Our study shows clear seasonality in the epidemiology of leptospirosis: Health professionals and facilities should be prepared to test and treat more leptospirosis patients from February until May each year in New Caledonia. Young men in the active age groups pay the heaviest toll to leptospirosis and should be the target of information and prevention campaigns. Improved and better targeted prevention messages are needed to avert human leptospirosis, avoidable deaths and related healthcare costs.

As in the French West Indies and New Caledonia, molecular diagnosis (q PCR) should be made readily available to tropical and subtropical resource-limited countries, including PICTs, who pay a heavy toll to this neglected disease [6]. This should improve ease of diagnosis and provide accurate, comparable and reproducible epidemiological surveillance data to inform local health authorities.

Leptospirosis has already been associated to the education level in urban slums [4]. Here, we report an age-specific pattern of leptospirosis seasonality and serogroups involved. Interestingly, this age-specific patterns suggest an association between school attendance and decreased exposure to an environmental risk of leptospirosis, an epidemiological pattern already observed in sub-tropical Japan [36]. Seasonal protection against leptospirosis by school attendance in New Caledonia is likely an unexpected benefit of schooling that deserves to be further investigated by *ad hoc* epidemiological investigations of exposure in school-age children. This mechanism could be further reinforced by timely health education and awareness messages in classrooms before the holidays during which children are exposed to environment-borne leptospirosis. Confirming this finding would also provide an unexpected yet startling evidence of how school education may contribute to reaching the Sustainable Development Goals in vulnerable populations of Oceania [49].

## Supporting information

S1 File Raw surveillance data. (XLSX)

S2 File Poisson statistics for age-specific seasonal incidence rates. (PDF)
