# Supplementary material for: Age-specific epidemiology of human leptospirosis in New Caledonia, 2006-2016
Source: PLoS One. 2020 Nov 30;15(11):e0242886. doi: 10.1371/journal.pone.0242886 (PMC7703958; doi:10.1371/journal.pone.0242886)
Supplement: S2 File — (PDF) [file pone.0242886.s002.pdf]

Poisson statistics for age-specific seasonal incidence rates

These calculations were made comparing

- 2 age groups: younger than 15 y.o. (as a proxy for school age) vs 15 y.o. and older,
- 2 seasonal periods: January and February (corresponding to infection during Austral summer holidays) vs March to December

Poisson statistics were computed using Stata and are summarized below:

**Age category**	**[0-15[**	**15 & older**			
Cases during schoolyear	61	625			
Cases during holidays	42	176			
Population (2014 census, https://www.isee.nc/)	63,698	205,069			
Time exposed during schoolyear (days)	306	306			
Time exposed during holidays (days)	59	59			
Person-days exposure during schoolyear	19,491,588	62,751,114			
Person-days exposure during holidays	3 758 182	12 099 071	**Risk ratio school children vs others**		**Mid-p**
Poisson schoolyear incidence rate p. 100,000 [95%CI]	0.31 [0.24-0.40]	1 [0.92-1.08]	kids vs. others during schoolyear	0.31	<0.0001
Poisson holiday incidence rate p. 100 000 [95%CI]	1.57 [1.22 - 2.03]	1.45 [1.25-1.69]	kids vs. others during holidays	1.08	0.1197
